# Índices antropométricos, actividad física y patrones alimentarios en estudiantes de nutrición y dietética de Colombia

**DOI:** 10.15446/rsap.V26n4.114092

**Published:** 2024-07-01

**Authors:** Maritza Sora-Gutiérrez, Gloria C. Deossa-Restrepo, Cristian D. Santa, Difariney González

**Affiliations:** 1 MS: Nut. Diet. M. Sc. Ciencias de la Alimentación y Nutrición Humana. Escuela de Nutrición y Dietética, Universidad de Antioquia. Medellín, Colombia. sandra.sora@udea.edu.co Universidad de Antioquia Escuela de Nutrición y Dietética Universidad de Antioquia Medellín Colombia sandra.sora@udea.edu.co; 2 GD: Nut. Diet. Esp. y M.Sc. Ciencias de la Alimentación y Nutrición Humana. Escuela de Nutrición y Dietética, Universidad de Antioquia. Medellín, Colombia. gloria.deossa@udea.edu.co Universidad de Antioquia Escuela de Nutrición y Dietética Universidad de Antioquia Medellín Colombia gloria.deossa@udea.edu.co; 3 CS: Estad. M. Sc. Estadística. Escuela de Nutrición y Dietética, Universidad de Antioquia. Medellín, Colombia. cdavid.santa@udea.edu.co Universidad de Antioquia Escuela de Nutrición y Dietética Universidad de Antioquia Medellín Colombia cdavid.santa@udea.edu.co; 4 DG: Estad. Ph. D. Educación. Facultad Nacional de Salud Pública, Universidad de Antioquia. Medellín, Colombia. difariney.gonzalez@udea.edu.co Universidad de Antioquia Facultad Nacional de Salud Pública Universidad de Antioquia Medellín Colombia difariney.gonzalez@udea.edu.co

**Keywords:** Índice de masa corporal, índice cintura/talla, actividad física, patrones alimentarios *(fuente: DeCS, BIREME)*, Body mass index, waist/height ratio, physical activity, eating patterns *(source: MeSH, NLM)*

## Abstract

**Objetivo:**

Analizar los cambios en la composición corporal (índice de masa corporal e índice cintura/talla), la actividad física y los patrones alimentarios en estudiantes de nutrición y dietética de universidades colombianas, entre los años 2017 y 2018.

**Materiales y Métodos:**

Estudio descriptivo longitudinal, con datos secundarios correspondientes al estudio multicéntrico de estudiantes de nutrición y dietética de universidades de Colombia y México. Muestra de 228 estudiantes de universidades colombianas, entre 18 y 31 años, evaluadas en dos momentos, con diferencia de un año. Incluyó variables sociodemográficas, antropométricas, de actividad física y consumo de alimentos. Se aplicó estadística descriptiva y análisis inferencial. Los patrones alimentarios se determinaron mediante análisis factorial confirmatorio.

**Resultados:**

El índice de masa corporal y el índice cintura/talla presentaron diferencias estadísticamente significativas entre las evaluaciones 1 y 2 (p<0,05), con aumento del riesgo cardiovascular, sin diferencias significativas en la actividad física entre las dos evaluaciones (p>0,05). Se determinaron 3 patrones dietéticos: a) cardioprotectores, b) altos en azúcar y c) altos en grasa, sin cambios entre evaluaciones.

**Conclusión:**

El presente estudio evidenció aspectos críticos en la alimentación, la composición corporal y la actividad física de las estudiantes, que se mantienen o empeoran en el tiempo, a pesar del avance en su formación académica en el área de alimentos, dietética y salud.

## Abstract (en)

**Objective:**

To analyze changes in body composition (Body Mass Index and Waist/Height Index), physical activity and eating patterns in Nutrition and Dietetics students from Colombian universities, between 2017 and 2018.

**Materials and Methods:**

Study longitudinal descriptive. With secondary data corresponding to the multicenter study of Nutrition and Dietetics students from universities in Colombia and Mexico. Sample of 228 students from Colombian universities, between 18 and 31 years old, evaluated at two moments with a difference of one year. It included sociodemographic, anthropometric, physical activity and food consumption variables. Descriptive statistics and inferential analysis were applied. Eating patterns were deter-mined by confirmatory factor analysis.

**Results:**

The Body Mass Index and the Waist/Height Index presented statistically significant differences between evaluations 1 and 2 (p<0.05), with increased cardiovascular risk; without significant differences in physical activity between the two evaluations (p>0.05). Three dietary patterns were determined: a) cardioprotective, b) high in sugar and c) high in fat, with no changes between evaluations.

**Conclusion:**

The present study showed critical aspects in the nutrition, body composition and physical activity of the students, which are maintained or worsen over time, despite the progress in their academic training in the area of food, dietetics and health.

El aumento en la prevalencia de exceso de peso en adultos jóvenes entre 18 y 29 años, producto de dietas poco saludables y sedentarismo, se considera un factor de riesgo importante para la aparición de enfermedades no transmisibles (ENT). Según el observatorio de la Organización Mundial de la Salud (OMS) en 2016, el sobrepeso y la obesidad en el mundo, en adultos de 18 años y más, fue de 39,1%, siendo un poco más alto en mujeres (39,7% vs. 38,5%) 1. En Colombia, para el año 2015, según la Encuesta Nacional de la Situación Nutricional 2015 (ENSIN), el exceso de peso en la población joven, de 18 a 22 años, fue de 27,5%, más alto en mujeres que en hombres (31,6%; 23,5%), y en el rango de 23 a 27 años de 43,2%, de nuevo mayor en mujeres (45,0% vs. 41,3%) 2.

Estudios en universitarios han demostrado que estos no advierten los riesgos en salud como algo cercano a ellos; además, que la educación formal que se brinda a profesionales de la salud no influye en la modificación de prácticas de riesgo, como sedentarismo, consumo de alcohol y alimentación inadecuada, las cuales favorecen el aumento del sobrepeso y la obesidad, que a su vez se asocian con riesgos de enfermedades cardiovasculares (ECV) 3-7. Algunos de estos resultados se observan en las áreas de la salud, a la que pertenece la carrera de Nutrición y Dietética (NyD) 8.

De otro lado, los hábitos alimenticios y la actividad física se relacionan de manera directa con el índice de masa corporal (IMC) 9-10. Los patrones de alimentación y la calidad global de la dieta determinan el riesgo de ENT, más que los nutrientes o los alimentos de manera particular 11. Al respecto, estudios llevados a cabo en universitarios chilenos encontraron un bajo consumo de alimentos saludables como pescado, leguminosas, frutas, verduras y alimentos integrales; además, un elevado consumo de *snacks* salados, bebidas azucaradas y alcohol 9. Resultados similares reportaron De Piero *et al.,* en universitarios de Tucumán (Argentina) 12. Así mismo, en un estudio longitudinal con estudiantes de Rumania, se encontró un aumento en el consumo de bebidas azucaradas y comidas rápidas; se reportó consumo diario de *snacks* y solo un consumo diario de frutas y verduras en 20% de ellos 10. Otros estudios en universitarios de países latinoamericanos han mostrado resultados similares 13-16.

Por otra parte, la valoración de la composición corporal se ha enfocado en el uso del IMC, junto con el perímetro de cintura (PC); no obstante, dicho índice como estimador del peso global presenta limitaciones en la valoración del estado nutricional 17, pues no permite establecer cómo se encuentra la composición corporal y, sobre todo, la distribución de grasa. Los índices asociados con adiposidad central se correlacionan con parámetros analíticos metabólicos relacionados con el riesgo de ECV, siendo el índice cintura/talla (IC/T) uno de los que presentan mayor sensibilidad para predecir la incidencia de riesgo cardiometabólico, no solo en personas con IMC y PC elevados, sino también en quienes presentan normalidad en dichos parámetros 18.

Se considera relevante conocer los cambios en la composición corporal, la actividad física (AF) y los patrones de consumo de alimentos en mujeres matriculadas en programas de nutrición y dietética en Colombia, puesto que son ellas las futuras profesionales que deberán promover estilos de vida y de alimentación saludable en la población. Lo anterior permitirá orientar las acciones, tanto curriculares como de bienestar universitario, que contribuyan a establecer estrategias que fomenten la cultura del autocuidado y la salud, en el marco de las universidades saludables.

Por lo anterior, este estudio se enfocó en analizar los cambios en la composición corporal (IMC e IC/T), la AF y los patrones alimentarios en estudiantes de NyD de universidades colombianas, entre los años 2017 y 2018.

## MATERIALES Y MÉTODOS

Estudio descriptivo longitudinal con dos momentos de valoración de datos (2017-1 y 2018-1). La investigación se realizó a partir de datos secundarios correspondientes al estudio multicéntrico de estudiantes de NyD de universidades de Colombia y México 19, cuyo objetivo fue determinar las prácticas alimentarias y los estilos de vida de riesgo para la salud y la nutrición de las estudiantes de NyD. Fue un estudio descriptivo transversal, en el que se evaluaron 583 mujeres matriculadas en el programa de NyD de cuatro universidades colombianas (n=439, 75,3%) y dos mexicanas (n=144, 24,7%). La información se recolectó mediante un formulario de Google Forms autodiligenciado. Se registró la frecuencia (días/semana), la duración (en horas) y el tipo de AF. Se reportó la frecuencia de consumo de alimentos de los últimos seis meses (diario, semanal, quincenal, ocasional, nunca); solo los grupos de grasas saturadas, monoinsaturadas y poliinsaturadas tuvieron ejemplos de los alimentos incluidos. Personal entrenado en el protocolo de la Sociedad Internacional de Cineantropometría (ISAK) realizó la evaluación antropométrica.

Para el presente estudio, se seleccionó una submuestra de 228 mujeres, con edades entre los 18 y los 31 años, que tenían datos completos de ambas mediciones, pertenecientes a cuatro universidades colombianas (Antioquia y Católica de Oriente (de Antioquia), Metropolitana (de Barranquilla) y del Sinú (de Cartagena)).

### Variables sociodemográficas

La edad, la universidad y el semestre matriculado.

### Variables antropométricas

Peso corporal, estatura y perímetro de cintura. El IMC se clasificó según los puntos de corte definidos por la OMS, así: bajo peso (<18,5 kg/mm), normalidad (18,5 a 24,9 kg/mm), sobrepeso (25,0 a 29,9 kg/m2) y obesidad (>30,0 kg/mm) 20, y el IC/T (circunferencia de cintura (cm)/ talla (cm), como riesgo cardiovascular (RCV) incrementado, cuando el valor era >0,50, y bajo <0,50 21.

### Estilo de vida

Se clasificó según las recomendaciones de la OMS, como físicamente activas (≥ 2,5 horas/semana de AF) y poco activas (<2,5 horas/semana de AF), de acuerdo con la frecuencia y duración de la AF 22

### Frecuencia de consumo de alimentos

Las categorías originales del cuestionario de frecuencia simple de consumo se reagruparon en diario, frecuente (semanal y quincenal) y poco frecuente (ocasional y nunca). Se incluyeron los siguientes grupos: carnes altas en grasa, cereales integrales, grasas saturadas (mantequilla, tocineta, coco y aceite de palma), grasas monoinsaturadas (aguacate, aceite de canola y oliva), grasas poliinsaturadas (aceite de soya, girasol y margarinas suaves), frutas, verduras, nueces y semillas, azúcares y dulces, chocolatinas, aguapanela, gaseosas y refrescos azucarados, productos fritos y bebidas alcohólicas.

### Aspectos éticos

Según los principios de la Declaración de Helsinki y del Ministerio de Salud de Colombia (Resolución n.o 008430 de octubre de 1993, Artículo 11), la investigación se clasificó como de riesgo mínimo. Asimismo, se garantizó la confidencialidad y el anonimato de la información recolectada. El Comité de Bioética de la Facultad de Odontología de la Universidad de Antioquia, mediante concepto n.o 88-2021, Acta n.o 07 del 20 de agosto de 2021, dio el aval y otorgó la aceptación ética para realizar el estudio. Es de anotar que la selección de las participantes para el estudio original fue aleatoria y la participación fue voluntaria, luego de firmar consentimiento informado.

### Análisis estadístico

Para el análisis descriptivo de las variables cualitativas se estimaron proporciones y frecuencias absolutas, y para los datos cuantitativos se utilizaron medianas y rangos intercuartílicos en el caso de que no se cumpliesen los criterios de normalidad, verificado con el test de bondad de ajuste Shapiro-Wilks. Se utilizó el *software* estadístico SPSS versión 27.

Se llevó a cabo un análisis factorial exploratorio (EFA) en los 15 grupos de alimentos de las tomas 1 y 2, con el fin de evaluar las cargas factoriales y definir los patrones alimentarios. Se hizo el análisis de consistencia interna para evaluar el grado de interrelación de los ítems del cuestionario de consumo simple y determinar si generaban resultados similares para el constructo general; se construyó la matriz de correlaciones policóricas, de acuerdo con la naturaleza ordinal de las respuestas de los ítems, para evaluar la medida de adecuación del muestreo con el método Kaiser-Mayer-Olkin (KMO), donde se definió como criterio de adecuación un valor de 0,7, y para evaluar la consistencia interna se utilizó el Alpha de Cronbach.

Las respuestas de los ítems del cuestionario se cuantificaron de 1 a 3 (1=diario, 2=frecuente, 3=poco frecuente), y para el EFA se definieron tres dimensiones de los patrones alimentarios (cardioprotectores, altos en grasas, y altos en azúcar), teniendo en cuenta la rotación varimax y el método de estimación por máxima verosimilitud (ML). Esto permitió establecer un modelo teórico de los patrones alimentarios, a partir de las frecuencias de consumo de los alimentos, con el fin de realizar un análisis factorial confirmatorio (CFA) para los resultados de las tomas 1 y 2. El CFA se ajustó por ML con el método de optimización no linear *nlminb,* se consideraron pesos factoriales adecuados mayores a 0,3 y se conservó la equivalencia del ajuste en ambos modelos, para poder realizar las comparaciones de estos con el test chi cuadrado.

Se extrajeron los puntajes *(score)* de los tres patrones alimentarios para ambos modelos y se compararon mediante el test de Wilcoxon para muestras pareadas, con el método de corrección de valores-p Benjamini-Hockberg, a fin de detectar diferencias en los patrones de consumo. Para interpretar el comportamiento de los puntajes de los patrones alimentarios, se estimaron las medianas de los puntajes con las categorías de frecuencia de consumo de cada ítem, donde puntajes negativos se asociaron con mayor frecuencia de consumo de alimentos y puntajes positivos con una menor frecuencia.

Todos los análisis relacionados con consumo de alimentos se hicieron con el *software* estadístico R V4.0.5, utilizando los paquetes *lavaan* para el CFA y *pysch* para EFA, consistencia interna y medida de adecuación.

## RESULTADOS

Participaron 228 estudiantes de sexo femenino, procedentes de las universidades de Antioquia (46%), Metropolitana (46%), Católica de Oriente (7,6%) y del Sinú (0,4%); al momento de la primera evaluación (Ev1) cursaban entre el primero y el octavo semestre, con mayor representatividad de los semestres 4, 6 y 3 (19,3%, 18,9% y 15,8%, respectivamente). En la segunda evaluación (Ev2) se encontraban entre el tercero y el décimo semestre, con más representatividad en el sexto y en el octavo (24,6% y 21,5%). El promedio de edad de la EV1 fue 20,7± 2,5 años y de la Ev2 fue 22,0±2,5 años.

La mayor parte de las estudiantes tenían adecuados el IMC (72,4% en Ev1 y 71,1% en Ev2) y el IC/T (87,7% en Ev1 y 85,5% en Ev2). Con respecto a la AF, un porcentaje elevado eran poco activas (46,9% en Ev1 y 46,5% en Ev2), seguido por una conducta sedentaria (29,8% en Ev1y 22,8% en Ev2). Las diferencias observadas entre Ev1 y Ev2 para las tres variables fueron estadísticamente significativas, lo que indica un aumento del sobrepeso y la obesidad evaluado por IMC, del riesgo de ENT, y un incremento en el porcentaje de personas activas (Tabla 1).

**Tabla 1:** Comportamiento del Índice de masa corporal, índice cintura/talla y actividad física, según las evaluaciones 1 y 2

	Evaluación 1		Evaluación 2		Valor p
	n	%	n	%	
Índice de masa corporal					<0,000*
Bajo peso	16	7,0	14	6,1	
Adecuado	165	72,4	162	71,1	
Sobrepeso	38	16,7	40	17,5	
Obesidad	9	3,9	12	5,3	
Índice cintura/talla					<0,000*
Adecuado	200	87,7	195	85,5	
Riesgo enfermedad cardiovascular	28	12,3	33	14,5	
Actividad física					<0,000*
Sedentarios	68	29,8	52	22,8	
Poco activos	107	46,9	106	46,5	
Físicamente activos	27	11,8	40	17,5	
Muy activos	26	11,4	30	13,2	

^*^ Diferencias estadísticamente significativas (prueba Chi cuadrado).

La mediana del IMC fue 22,04 ± 4,31 (Ev1) y 22,44 ± 4,32 (Ev2), con un incremento significativo (p<0,05). Respecto al IC/T, la mediana fue 0,44 ±0,05 (Ev1) y 0,45± 0,06 (Ev2), con diferencias significativas (p<0,05). Se presentó aumento del riesgo cardiovascular tanto por IMC como por IC/T en la segunda medición. Para la AF se observó una mediana de 67,50 ±135 minutos/semana (Ev1) y 90,00±135 minutos/semana (evaluación 2), sin diferencias significativas (p>0,05) (Tabla 2).

**Tabla 2:** Cambios en el índice de masa corporal, índice cintura/talla y actividad física en estudiantes universitarias, en dos periodos analizados

	Evaluación 1 Mínimo			Evaluación 2 Mínimo			Valor p
	Mediana	Mínimo	Máximo	Mediana	Mínimo	Máximo	
Índice de masa corporal	22,04	14,11	38,41	22,44	14,30	39,19	0,000*
Índice cintura/talla	0,44	0,34	0,68	0,45	0,36	0,67	0,003*
Actividad física (minutos/semana)	67,50	0	600	90,00	0	600	0,153

^*^ Diferencias estadísticamente significativas (prueba de rango de signos de Wilcoxon).

Al analizar la frecuencia de consumo simple de alimentos en ambas evaluaciones, se encontró que se conservaron los mismos tres patrones dietéticos. El patrón 1, denominado *cardioprotector,* se caracterizó por el reporte de un consumo mayor de leguminosas, cereales integrales, grasas monoinsaturadas y poliinsaturadas, frutas, verduras, nueces y semillas. El patrón 2, nombrado *altos en azúcar,* incluyó azúcares y dulces, chocolatinas, agua de panela, gaseosas y refrescos azucarados, mientras que el patrón 3, nombrado como altos en grasa, incluía carnes altas en grasa, grasas saturadas, productos fritos y bebidas alcohólicas (Tabla 3).

**Tabla 3:** Pesos factoriales de los grupos de alimentos, según patrones alimentarios, en estudiantes de NyD de universidades de Colombia

	Evaluación 1			Evaluación 2		
Grupo de alimentos	Cardio protectores	Patrones de alimentación altos en azúcar	Altos en grasas	Cardio protectores	Altos en azúcar	Altos en grasas
Leguminosas	0,317			0,286		
Cereales integrales	0,458			0,367		
Grasas monoinsaturadas	0,361			0,528		
Frutas	0,729			0,673		
Verduras	0,599			0,601		
Nueces y semillas	0,358			0,362		
Grasas poliinsaturadas	0,300			0,344		
Azúcares y dulces		0,504			0,481	
Chocolatinas		0,354			0,545	
Agua de panela		0,372			0,407	
Gaseosas y refrescos azucarados		0,382			0,571	
Carnes altas en grasa			0,369			0,467
Grasas saturadas			0,452			0,489
Productos fritos			0,707			0,481
Bebidas alcohólicas			0,154			0,415

No se identificaron diferencias estadísticamente significativas entre las dos evaluaciones para ninguno de los patrones de consumo encontrados (cardioprotectores, p=0,772; altos en azúcares, p=0,808 y altos en grasa, p=0,503). 

## DISCUSIÓN

Este estudio permitió conocer la tendencia en el comportamiento de algunos indicadores antropométricos que reflejan el estado nutricional, como son el IMC y el IC/T, al igual que el comportamiento de la AF y los patrones de consumo de alimentos en mujeres estudiantes de NyD de Colombia.

Los resultados en el IMC indican que la mayoría de las estudiantes tenía un peso adecuado en ambas evaluaciones. Algunos estudios han reportado hallazgos que concuerdan con estos resultados. Al respecto, Durán-Agüero *et al.* hicieron una investigación en estudiantes de primero a cuarto año de NyD en Chile, en su mayoría mujeres, y reportaron normopeso según IMC en la mayoría de ellas 23; de igual forma, Rangel *et al.,* en su estudio con universitarios de las áreas de la salud de Bucaramanga (Colombia), encontraron que la mayoría de las mujeres tenía un IMC adecuado 24.

En el presente estudio se observó un aumento significativo en el IMC entre la Ev1 y Ev2, lo que se relaciona posiblemente con un incremento de la grasa corporal. Al respecto, existen pocos estudios que utilizan un diseño de medidas repetidas para evaluar cambios en dicho indicador en estudiantes universitarias, específicamente de NyD, y hay pocos estudios en universitarios de diferentes áreas, incluyendo las de salud, que reportan resultados similares, evaluando el IMC en dos momentos 25,26. Adicionalmente, se observó un aumento en el exceso de peso (sobrepeso y obesidad) en la segunda evaluación, lo cual se convierte en un factor de riesgo importante para el desarrollo de ENT. Yaguachi *et al.* también observaron un aumento en la prevalencia de exceso de peso medido por IMC en universitarios, transcurridos tres años académicos, y así mismo, aumento en el porcentaje de grasa corporal 27. A diferencia de estos hallazgos, Soto Ruiz *et al.,* en un estudio realizado en la Universidad de Navarra-Pamplona, con estudiantes de diferentes carreras, no encontraron diferencias significativas en el sobrepeso y la obesidad, transcurrido un periodo de dos años 28. El aumento en el IMC, y específicamente el exceso de peso, puede estar influido por el consumo frecuente de alimentos altos en grasa y bebidas azucaradas. Al respecto, Yaguachi *et al.* encontraron una relación de comidas rápidas y gaseosas con el aumento de adiposidad central e incremento del peso corporal 27. Otro factor que posiblemente influye en el IMC es un bajo nivel de AF. Huaman *et al.* EncontraroB n una relación inversa entre el IMC y la AF 29.

En cuanto al IC/T, indicador que permite evaluar la adiposidad abdominal y su asociación con el riesgo de enfermedades cardiovasculares, se encontró que, en los dos momentos, las medianas indicaron un riesgo bajo de ENT; sin embargo, su comportamiento fue similar al del IMC, con un incremento estadísticamente significativo en la segunda evaluación. El aumento de este, aun por debajo de los puntos de corte, indica un incremento en la acumulación de grasa visceral, que a futuro podría generar riesgos para la salud, puesto que el aumento del tejido adiposo en personas obesas produce una mayor infiltración por macrófagos y células T CD8+, ocasiona un cambio en estas células, de modo que pasan de un estado antiinflamatorio a un estado proinflamatorio, con el consiguiente aumento de citoquinas (TNF- α, IL-6, MCP-1, IL-ip), que ocasiona un estado inflamatorio crónico de bajo grado, lo que se asocia con mayor riesgo de resistencia a la insulina, implicada en la diabetes mellitus tipo 2, la obesidad, el síndrome metabólico y la ECV 30. Al respecto, Bojórquez-Díaz *et al.* también describieron en estudiantes universitarios un promedio de IC/T inferior a 0,50, pero mayor (0,47) al obtenido en el presente estudio; además, analizaron la probabilidad de riesgo entre la presión arterial y la relación del índice cintura/ talla (IC/T), y encontraron que tener un valor alterado de relación IC/T (>0,5) aumentó tres veces la probabilidad de presentar hipertensión en mujeres 31. El aumento en el IC/T se ha asociado con los patrones alimentarios poco saludables, que incluyen consumo de alimentos altos en grasa saturada y alcohol y bajo consumo de frutas y verduras 32.

Con relación a la actividad física, un porcentaje elevado de las participantes se clasificaron como poco activas y sedentarias en ambas evaluaciones. Tanto Rangel *et al.* como Mazurek *et al.* obtuvieron resultados similares, relacionados con bajo nivel de actividad física en una alta proporción de estudiantes de nutrición y otras áreas de la salud 24,33. Por su parte, Durán-Agüero *et al.* encontraron un nivel de sedentarismo mayor (85,2%) 23. El mantener bajos niveles de AF en universitarios se ha asociado con falta de tiempo, que va en relación directa con las altas cargas académicas. En un estudio de Fortino *et al.,* el 64,6% de las estudiantes de licenciatura en Nutrición declaró haber reducido la AF al entrar a la universidad 13. Por su parte, Aceijas *et al.* encontraron que el 40% de los estudiantes refirió la falta de tiempo como la razón principal para no usar el gimnasio de la universidad 34. La práctica insuficiente de ejercicio físico en universitarios puede generar un mayor riesgo para el incremento del peso y la grasa corporal, y con el ello el riesgo de ENT 22.

Con respecto al consumo de alimentos, su análisis permite caracterizar los estilos de vida y definir posibles factores de riesgo con relación a la ingesta frecuente de alimentos que contienen nutrientes o sustancias de riesgo que pueden alterar la salud, o un consumo insuficiente de alimentos con nutrientes protectores para la prevención de enfermedades prevalentes en el medio, como son las ENT. En este sentido, definir las tendencias hacia el consumo de ciertos alimentos permite caracterizar los patrones de consumo que predominan en los grupos de población. En las estudiantes de NyD colombianas se encontraron tres patrones, que tuvieron un comportamiento similar en las dos evaluaciones: un patrón de alimentos cardioprotectores, ricos en nutrientes como ácidos grasos monoinsaturados, y otros compuestos como fibra soluble e insoluble y antioxidantes, y dos patrones poco saludables que indican un consumo mayor de alimentos altos en grasas y otro en azúcares. Al respecto, un estudio realizado en estudiantes de sexo femenino, de enfermería, educación física y farmacia de Brasil 35, encontró que una proporción mayor al 50% consumía frutas, verduras y fríjoles cinco o más veces por semana; sin embargo, también reportaron factores de riesgo para ENT, asociados con consumo >5 veces/semana de productos altos en azúcar como los refrescos, y alimentos altos en grasa saturada como la leche entera y las carnes con grasa visible. En el grupo con sobrepeso reportaron una proporción menor en el consumo de gaseosas azucaradas y alimentos altos en grasa; no obstante, este aspecto debe tenerse en cuenta, ya que son personas que presentan varios factores de riesgo en salud.

El mantener determinados comportamientos alimentarios por parte de estudiantes universitarios, durante un periodo de tiempo académico, puede estar asociado con diversos factores como la disponibilidad de alimentos, la situación económica y el estado de ánimo generado por las presiones académicas o la influencia de los compañeros, tal como lo reportaron Kabir *et al.*^36^.

Lotrean *et al.* llevaron a cabo un estudio en estudiantes en una ciudad universitaria de Rumania, en el cual se definieron patrones alimentarios que fueron comparados en años posteriores, y cuyo resultado más relevante fue que pasados 13 años, aproximadamente, los patrones dietéticos eran bastante similares, y en el presente estudio se observó un resultado similar, puesto que, luego de transcurrido un año de estudio universitario, se encontraron los mismos tres patrones alimentarios. El patrón de carnes blancas, cereales y fibra, encontrado en universitarios de Rumania, comparte algunas características con el que se encontró en las universitarias de Colombia, relacionado con el consumo frecuente de frutas, vegetales, leguminosas, nueces y algunos cereales integrales. Una diferencia importante está en el patrón 3 de los estudiantes de Rumania, en el cual había un consumo mayor de alimentos altos en grasa y azúcares, mientras en Colombia se encontraron dos patrones diferenciados, uno indicando la tendencia hacia el consumo de grasas y otros al consumo de azúcares 10.

En las estudiantes con un patrón de consumo de alimentos cardioprotector, se generan beneficios en la prevención de ENT, relacionados con el efecto de cada uno de estos alimentos. El consumo de nueces y grasas ricas en ácidos grasos monoinsaturados tiene efecto en la disminución del colesterol LDL y aumento del colesterol HDL. Las leguminosas contienen fitoquímicos bioactivos como fitoesteroles y saponinas, que reducen la absorción intestinal de colesterol. Para los cereales integrales, se destaca su efecto sobre el control glucémico y las cifras de colesterol total y LDL, relacionado con su contenido de fibra. Las frutas y las verduras son ricas en compuestos fenólicos y en vitaminas A y C, y contienen antioxidantes útiles en la protección cardiovascular 37.

Patrones alimentarios poco saludables, como el consumo de alimentos altos en grasa o altos en azúcar, generan factores de riesgo para ECV. Un patrón de alimentos altos en grasa saturada se ha asociado con un mayor grado de adiposidad visceral 32, la cual genera mayor predisposición a resistencia a la insulina, ECV y síndrome metabólico 38. Por su parte, el patrón de alimentos altos en azúcar aumenta el riesgo de obesidad, dado su bajo poder saciante y alta palatabilidad, que induce a un consumo elevado 37. Además, puede generar un aumento de los triglicéridos y la glucosa, además de un mayor riesgo potencial de ECV en el largo plazo 38.

### Limitaciones

Una de las limitaciones importantes está relacionada con el uso de un cuestionario de consumo de alimentos, basado solo en la frecuencia simple de consumo de alimentos, que puede tener menor precisión en el análisis de consumo si se compara con otros métodos como el recordatorio de 24 horas o la frecuencia de consumo semicuantitativa.

Otro aspecto por considerar es que no se incluyó una muestra de estudiantes de ambos sexos, puesto que no se logró tener un tamaño de muestra consistente de hombres de esta carrera en el estudio de base. Sin embargo, es clara la importancia de estudiar las diferencias por sexo.

El tamaño de la muestra se vio limitado por la pérdida de sujetos en la segunda evaluación, por el tiempo transcurrido de un año.

### Fortalezas

Tener disponibles datos de estudiantes universitarias colombianas, evaluadas en dos periodos, permitió desarrollar un estudio longitudinal, que brinda información valiosa para analizar y generar acciones de prevención de ENT que se pueden implementar desde el currículo y desde los programas de bienestar universitario.

Aunque el cuestionario de frecuencia de consumo de alimentos fue cualitativo, consideró diferentes grupos de alimentos y cinco opciones de respuesta, que permitieron identificar tendencias en dicha frecuencia de consumo. Además, fue diligenciado por estudiantes de NyD que desde sus primeros semestres están familiarizadas con este instrumento.

Son escasos los estudios publicados acerca de los cambios que se presentan en indicadores de composición corporal, actividad física y patrones alimentarios que están asociados con el riesgo de ENT en estudiantes de NyD.

El presente estudio logró evidenciar aspectos críticos en la alimentación, la composición corporal y la AF de las estudiantes participantes, que se mantienen o empeoran en el tiempo, a pesar del avance en conocimientos que logran en el área de alimentos, dietética y salud. Además, el análisis de alimentos permitió identificar patrones de alimentación cardioprotectores que se relacionan con un menor riesgo cardiovascular, pues se asocian de manera inversa con IMC e IC/T.

Es claro que el conocimiento y la educación en el área nutricional constituyen un componente importante en las intervenciones que buscan mejorar los estilos de vida, no obstante, es posible que existan otros condicionantes igual o más relevantes por intervenir para lograr los cambios y una mejoría que lleven a una verdadera promoción de la salud. En el transcurso de un año de vida universitaria se pueden ir acumulando factores de riesgo asociados con un patrón de alimentación poco saludable, aumento del peso relacionado con incremento de la grasa corporal a nivel abdominal, prácticas de actividad física insuficientes y posiblemente otros factores que en el presente estudio no fueron analizados, como es el caso de los datos bioquímicos, que podrían ser de interés para lograr un análisis más completo de la situación. La complejidad de esta situación pone en riesgo la salud y la calidad de vida de esta población, que además se está formando en un área de la salud en la cual se promueven estilos de vida saludables, con el fin de reducir el impacto que tienen las ENT en la morbimortalidad y, más allá de las palabras, el ejemplo convence y motiva.

Es importante considerar que las propuestas de intervención para promover estilos de vida saludables, que hacen las universidades desde de los programas de bienestar, deben estar coordinadas con las dinámicas universitarias, es decir, con los espacios de esparcimiento, las cargas académicas, el manejo del estrés, la evitación de consumo de licor y tabaco, la actividad física, el tiempo libre (incluyendo el sueño) y la oferta de productos alimenticios saludables y a precios accesibles, entre otros, con el fin de lograr un mayor impacto en el bienestar general de la población universitaria ♣ 
